# Editorial: Nanomaterials for Communicating With the Brain

**DOI:** 10.3389/fnins.2022.918949

**Published:** 2022-05-02

**Authors:** Wei Tong, Michael R. Ibboston, Diego Ghezzi

**Affiliations:** ^1^School of Physics, The University of Melbourne, Parkville, VIC, Australia; ^2^National Vision Research Institute, Australian College of Optometry, Carlton, VIC, Australia; ^3^Department of Optometry and Vision Sciences, School of Health Sciences, The University of Melbourne, Parkville, VIC, Australia; ^4^Center for Neuroprosthetics and Institute of Bioengineering, School of Engineering, École Polytechnique Fédérale de Lausanne, Geneva, Switzerland

**Keywords:** neural stimulation, neural recording, nanomaterials, implantable, neural interface

## Introduction

Neural interfacing technologies that allow a degree of control over brain function via stimulation and/or recording of brain activity are beginning to have a significant role in medicine. The bionic ear, deep brain stimulation, control of robotic limbs and prosthetic vision devices represent some of the current applications. In basic neuroscience research, neural interfaces are important tools for understanding complicated neural circuits and functions, and they are essential for understanding complex neural processes, such as those that generate memory, perception, cognition, and behavior. To realize the full potential of neural interfacing technologies, their continuous development requires that the devices work safely, stably, reliably, and efficiently over long periods of time.

The materials that directly interact with neurons via stimulation or recording are the core components of neural interfaces. The performance and longevity of the devices are largely dependent on the materials from which they are built. The interfacing materials have therefore become a major focus of development in this rapidly expanding field. A long-term, high-performance neural interface will need to be manufactured from interfacing materials that can function stably and reliably for long periods, evoke minimum inflammatory foreign body responses, and have the potential to modulate or sense neural activities at high temporal and spatial resolutions. It is extremely difficult to find a material that can fulfill all of these requirements, but recent advances in functional nanomaterials have made it possible to address these challenges, and the future appears bright.

In this Research Topic, we selected a total of five publications, with contributions from over 40 world-leading and emerging researchers, on the basis of a thorough peer review process with multiple iterations of manuscript revisions. These publications have a combination of formats including Original Research, Review and Mini Review. They cover a variety of materials, and review and report progress in different fields such as materials synthesis, electrode fabrication and characterization, and biological assessment.

## Electrical Communication

Despite its long development history, electrical stimulation and recording remain the most used techniques for communicating with the brain. Novel materials continue to emerge and are being employed, aiming at solving the challenges in electrode design and fabrication (Wellman et al., 2018). Conventional devices fabricated with metal electrodes have limited electrochemical properties. The low charge injection capacities of these electrodes limit the amount of charge that can be safely delivered to the tissue for evoking neural responses, and their high electrochemical impedances result in low signal-to-noise levels during neural recording. The lifetimes of the conventional devices are restricted due to the instability of the electrode materials, as well as the adverse tissue responses associated with implantation. These adverse tissue responses are induced during the insertion process and because of the chemical and mechanical mismatch between the tissue and electrodes.

To overcome the above issues, current trends are to use thinner and more flexible structures, together with electrode materials that have improved electrochemical properties (Zhang et al., 2021). In this Research Topic, Hejazi et al. reviewed the progress in carbon-based microfiber electrode development. These microfiber electrodes, with ultrathin and flexible structures, are optimized with enhanced electrochemical properties, which have proved to be useful for long-term implantation. Coincidently, Fu et al. performed various measurements and systematically compared the properties of different carbon-based microfibers. They then proposed a new strategy to improve the stability of electrode insulation without sacrificing the flexibility of the electrode materials.

While some frontline research pursues innovative electrode structures, others have successfully used more conventional designs, which immediately benefit from mature surgical implantation techniques. Without altering device configuration, these studies have shown that electrode performance can be improved using advanced coating materials that possess better biocompatibility, stability, and electrochemical and mechanical properties. One example is demonstrated by Hyakumura et al., in which the authors examined a new type of conductive hydrogel as a coating for improving platinum electrodes for deep brain stimulation. From chronic *in vivo* assessment, the optimized coating was demonstrated to have better mechanical and electrochemical properties, as well as stability.

## Optical Communication

In addition to electrical communication, researchers are developing techniques that can modulate neural activities via light (Thompson et al., 2014). Compared to electrical stimulation, optical stimulation techniques are thought to be beneficial because they can provide more selective stimulation and higher spatial resolution, while also offering less invasiveness than implanted electrodes. While optogenetics is now a routine technique in neuroscience laboratories and clinical trials are underway for vision restoration, they require genetic modification. Opsins that can provide high light sensitivity, fast kinetics and are able to respond to longer excitation wavelengths are still under development. Alternatively, interest in photoactive materials that can convert light into heat or electric fields for neural stimulation has increased dramatically. Without the need for genetic modification, many of these photoactive materials are sensitive to wavelengths in the near-infrared range, which can penetrate deeper into neural tissue than the visible light wavelengths used by optogenetics. In the format of nanoparticles, they are considered much less invasive than implanted devices. The current Research Topic includes a work about quantum dots for optical stimulation of neurons, in which Karatum et al. synthesized InP/ZnS core/shell nanoparticles. The heterostructure presented by the authors was found to generate improved photo-responses and was also found to be safe and effective for stimulating cultured neurons.

## Mechanical Communication

As neurons are sensitive to mechanical stimuli, different forms of mechanical stimulation have been employed to modulate neural activity. For example, therapeutic delivery of ultrasound to the brain for treating brain cancers and other neurological disorders started in the 1950 (Blackmore et al., 2019). Recent years have seen the exploitation of piezoelectric materials in neural engineering (Marino et al., 2017). These piezoelectric materials are able to convert mechanical into electrical energy and *vice versa*, and they have been suggested as possible wireless and non-invasive approach for neural stimulation. Peng et al. reviewed the progress that has been made using surface acoustic wave devices fabricated using piezoelectric materials for neural stimulation. The authors discussed the potential mechanisms and summarized the preliminary successes published so far. More research is expected in the future on the use of piezoelectric materials for neuromodulation, so this review is a useful starting place to gain an understanding of the field.

## Final Remarks

The ultimate goal of many research efforts in developing these interfacing materials is to translate them into medical practice. While *in vitro* and short-term *in vivo* studies are useful for demonstrating the functions of the newly developed materials, we expect to see considerable ongoing research to assess their chronic, long-term performance. The development of neural interfacing technologies requires multidisciplinary efforts and is likely to be accelerated through expanding collaborations.

## Author Contributions

All authors listed have made a substantial, direct, and intellectual contribution to the work and approved it for publication.

## Funding

WT was supported by a University of Melbourne Early Career Researcher Grant (2021ECR091) and a Linkage Grant from the Australian Research Council (LP180100638).

## Conflict of Interest

The authors declare that the research was conducted in the absence of any commercial or1 financial relationships that could be construed as a potential conflict of interest.

## Publisher's Note

All claims expressed in this article are solely those of the authors and do not necessarily represent those of their affiliated organizations, or those of the publisher, the editors and the reviewers. Any product that may be evaluated in this article, or claim that may be made by its manufacturer, is not guaranteed or endorsed by the publisher.
